# Humour uncovers the wide landscape of life: From laughter to freedom

**DOI:** 10.1038/s44319-025-00494-6

**Published:** 2025-06-03

**Authors:** Vladimir Leksa

**Affiliations:** Laboratory of Molecular Immunology, Institute of Molecular Biology, Slovak Academy of Sciences, Bratislava, Slovakia

**Keywords:** Economics, Law & Politics, Evolution & Ecology, Neuroscience

## Abstract

Humour is an important tool to maintain our physical and mental health, cultivate our marriages and relationships, build open societies, keep the peace, and defend against madness and evil.

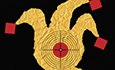

“Of all the bodily movements which at once shake the body and soul, laughter is the healthiest, because it promotes digestion, circulation, evaporation, and refreshes the vital sap in all the organs.” Thus wrote Christoph Wilhelm Hufeland, personal physician of Johann Wolfgang Goethe and Friedrich Schiller. A hundred years later, comedian Groucho Marx said the same in different words: “A clown is like aspirin, only he works twice as fast.” They were both right. Similar to the physician and the comedian, science has concluded that humour has indeed a profoundly positive effect on the physical and mental health of a laughing individual (Savage et al, 2017). The caveat, however, is that this is only the case if humour does not humiliate others, but rather that we are able to make fun of ourselves.

“A clown is like aspirin, only he works twice as fast.”

## Humour at the dawn of culture

Poet John Keats once blamed physicist Isaac Newton for reducing our pleasure in looking at a rainbow by discovering that white light is actually a spectrum of colours. Yet, it’s just the opposite—the understanding of why we see the rainbow the way we do has led to an even better appreciation of the wonders and beauty of nature, as biologist Richard Dawkins put it in his book *Unweaving the Rainbow: Science, Delusion and the Appetite for Wonder* (1998). Similarly, scientific immersion in the laws of laughter cannot rob us of our sense of humour. However, science will never be able to explain all aspects of humour. It can, it is true, describe what humour does to us, all those physiological actions and biochemical reactions, but it will never give us the answer as to why exactly this joke makes one person laugh and not the other one, and why the third one never laughs at all. It’s like with the rainbow. The laws of optics may explain why we see it just so, but they will not explain why only some people write a poem about it.

… scientific immersion in the laws of laughter cannot rob us of our sense of humour.

But when did humour emerge in human society? According to comedian Miroslav Horníček, there were some natural entertainers among the cavemen who could comfort their cave-mates with funny stories during long, cold and dark nights. And the fellow residents laughed, slapping their bare thighs. Later, when people started wearing skirts and trousers, slapping thighs lost its acoustic effect. In order to somehow imitate it, listeners began to clap their hands together—that is how applause came about. According to this theory, we can say that humour arose at the beginning of human culture.

One way or another, the primary question remains what makes a person laugh. For an answer, we can go to the playwright, author of absurd dramas, philosopher, politician and humourist Václav Havel. In his essay *The Anatomy of a Gag* (1963), he notes that a laughter-inducing situation is usually based on a paradox: “When one is lamenting for the death of his wife, it is not a gag. When one is mixing gin-fizz, it is not a gag. But as soon as Chaplin gets the report that his wife has died, and he turns away from us, shakes himself in tears, and then slowly turns back to us, just to reveal that he was not shaking in tears, but instead mixing gin-fizz, it’s a gag.”

…and that’s when we burst out laughing. The subject will at first change facial expression in a way that was already described in the 19^th^ century by the French physiologist Guillaume–Benjamin Duchenne. We call it a smile (Gunnery and Ruben, 2016). Beware not to confuse it with a say-cheese smile. Unlike the latter, a mannered one, the former, a real ‘Duchenne smile’, involves all the muscles of the face, especially those innervated by the facial nerve, specifically in the eye and lip area.

Tears well up in the eyes of the affected person. With stronger seizures, colic occurs, the subject breaks forward at the waist holding the abdomen. As a rule, this condition is accompanied by loud phonetic manifestation without clear intonation and articulation. Rather than human speech, it is reminiscent of animal sounds: squealing, neighing, or hee-hawing. In other words, a smile turns into laughter or a bray. The length of this physiological reaction depends, first, on the intensity of the perception—that is, on the strength of the joke—and, second, on the mental predisposition of the subject—that is, on his or her sense of humour. The latter is the most important condition, because in its absence, even the best Chaplin gag will not make the subject laugh.

## Grand consilium on the compelling questions of humour

Experts may go into more depth. A neurologist would explain that laughter is controlled by the limbic system – the emotional centre of the brain; that neurons are responsible for transmitting excitation in the brain; and, finally, that neurons communicate with each other through molecules called neurotransmitters, such as serotonin, dopamine or endorphins, which are particularly important for the outbreak of laughter (Pearce, 2004). A biochemist would explain the chemical pathways of neurotransmitter synthesis, and describe where and how they bind, and what exactly they trigger when we laugh (Barenz et al, 2024), and an immunologist would add how important this is for our health (Sakai et al, 2013).

If we go on, we learn that laughter can also be a sign of illness. For example, one scientific study (Adour, 1989) claims that the most famous smile in history is nothing more than a symptom of so-called Bell’s palsy, a temporary paralysis of facial muscles, which mainly affects the cheeks of new mothers. According to its authors, Mona Lisa, who allegedly had been pregnant shortly before she sat for Leonardo da Vinci, suffered from this disorder.

And finally, a sociologist may come in to explain that a sense of humour has often been identified as a key factor improving the quality and strength of a partnership, provided that both have it (Brauer and Proyer, 2018; Lauer et al, 1990; Verstaen et al, 2020). It’s true, I have a friend whose marriage broke up because of his weird sense of humour. He convinced his wife to get their fish vaccinated against pneumonia. She filed for divorce as soon as she got back from the vet with the aquarium.

… a sociologist may come in to explain that a sense of humour has been often identified as a key factor improving the quality and strength of a partnership, provided that both have it.

## Are there genes for humour?

But are there genes that determine whether we have a sense of humour and what kind of joke make us laugh? In short, no even though there are enough individuals in which the complete absence of humour could be studied. Despite that a wide range of respondents are at disposal for research—from those who do not laugh at all to constantly laughing ones who are a refreshing addition to any company—the specific gene responsible for the sense of humour has not yet been identified. However, some gene polymorphisms were proposed to be associated with humour. By instance, the short variant of the promoter region (5-HTTLPR) of the transporter gene (SLC6A4) for the neurotransmitter serotonin was suggested to confer heightened susceptibility to jokes (Haase et al, 2015).

In addition, a variant of oxytocin, another important neurotransmitter, termed OXTR rs53576, has been shown to correlate with a good marriage (Monin et al, 2019) and hence, with a sense of humour as described above. Oxytocin, a peptide hormone released from the hypothalamus, plays a key role in uterine contractions during childbirth and is essential for the production of breast milk after childbirth. However, it affects men too. The more fathers play with their children, the more oxytocin they have (Scatliffe et al, 2019). Or vice versa? And finally, this ‘joy hormone’ also has positive effects on the social level, specifically in establishing and strengthening long-term relationships (Dunbar, 2022; Feldman, 2017; Love, 2014).

## Absence of humour as a sign of totalitarianism

A sense of humour could therefore have positive effects not only on the health of individuals or marriages, but also on society as a whole. Indeed, the ability to laugh at historical traditions is a sign of a free, developed, self-confident and healthy society. On the contrary, hypersensitivity to national and religious symbols, offensiveness, censorship, in short, the absolute absence of a sense of humour have always been unmistakable signs of unfreedom. Tyrants never had a sense of humour. They are “agelasts”, as François Rabelais called people refusing to laugh. At themselves, of course. Their motto was expressed by the monk Jorge de Burgos in the novel *The Name of the Rose*: “Laughter is a devilish wind which deforms the lineaments of the face.”

… hypersensitivity to national and religious symbols, offensiveness, censorship, in short, the absolute absence of a sense of humour have always been unmistakable signs of unfreedom.

The term agelast comes from the Greek word *agelastos*, which means “not laughing”. There is something in humour that does not agree with agelasts. Why? They simply hate paradoxes, that is, situations that cause laughter. In the world of the agelasts, everything is set and clear forever, without paradoxes. Their world is a totalitarian one. And after all, it is difficult for such people to live in peace, isn’t it? Hence, we can deduce that humour could therefore play a significant role in maintaining peace among nations.

The author of *The Name of the Rose*, semiotician Umberto Eco, in his essay entitled *How to Spot a Fascist* (1995), has distinguished several signs that occur in varying numbers and combinations in dictatorships but also in democracies, for example in the programmes of political parties, the doctrines of civil or religious movements, or even in the opinions of artists and philosophers. In his essay, he speaks of ur-fascism: eternal fascism, as the original prototype of every dictatorship. Among the characteristic features of ur-fascism, Eco included a cult of tradition, the rejection of progress, irrationalism and attacks on critical thinking. In totalitarianism, language becomes increasingly poorer in terms of vocabulary and more primitive in terms of syntax—simple slogans and challenges are important and sufficient.

Other distinguishing features of an existing or emerging dictatorship are loyalty to the leader, elitism, denial of diversity and the constant spread of a sense of threat, which gives rise to nationalism, xenophobia, racism, anti-Semitism, homophobia and a hierarchically organized society. The result is the suppression of human rights, oppression of minorities and the necessity of waging a permanent war.

To this list, I would add one more sign: the absence of a sense of humour. I do not mean the ability to humiliate others. On the contrary, the latter feature is hypertrophied in dictators. By a sense of humour, I mean the gift of self-irony, the ability of not taking ourselves seriously and being able to laugh at ourselves. In that sense, dictators are complete invalids.

Not surprisingly, making humour in dictatorships has always been dangerous. That is why Hugo Haas, Jan Werich, Jiří Voskovec, Kurt Tucholsky and many others had to flee from Nazi Germany; Joachim Ringelnatz was prohibited to perform in public. Ephraim Kishon, Miloš Forman, and many other artists had to flee from communist regimes while humourists generally did not live to old age in Russia.

Especially in Russia, making jokes was, and still is, dangerous not only for professional comedians and writers, but also for ordinary people. The famous Russian physiologist Vladimir Mikhailovich Bekhterev, the discoverer of ankylosing spondylitis (Bekhterev’s disease), an autoimmune disease of the spinal vertebrae, had a developed sense of humour, although he did not make a living from it. On the contrary, he might have died of it. It is said, that after visiting one of his patients, his colleagues asked him why he was coming so late. He, with his characteristic irony, replied: “I have just examined a paranoiac with a small, dry hand.” Since some of his colleagues had other character traits more developed at the expense of humour—envy, servility, slander—the dry-handed paranoid probably received the report the same day. Bekhterev died shortly after under unclear circumstances. The patient’s name was Stalin (Kesselring, 2011).

## There is always someone who gets offended

However, agelasts, humourphobic people, live not only in dictatorships. They have been at every place and time throughout history. Marcus Porcius Cato used to be a prominent Roman politician allegedly ending every speech in the Senate with the notion that Carthage must be destroyed. What is less well known is that in his position as chief censor he pursued actors. He was, as the famous comedian Jan Werich wrote about him in his book *The Smile of a Clown*, a man without humour.

Since, Carthage has been destroyed and censorship has sustained in many ways to this day. The radius of action of censors is unlimited, because there will always be someone who feels offended by a joke. Especially among people with power. Today, few of us will probably remember Mervyn Stockwood, and yet he was a highly respected person at the time: the Bishop of Southwark. After the premiere of the film *The Life of Brian*, Stockwood met with the film’s creators, members of the Monty Python group, on a BBC television programme. The bishop accused them of blasphemy and insulting Jesus Christ. In short, he was offended. Shortly thereafter, Rowan Atkinson, later known as Lord Blackadder or Mr. Bean, reprimanded the Bishop at the BBC for offending the worshippers of Monty Python. The bishop was wrong, the film did not insult Jesus. It mocks fanatics of all kinds. That is why it is still relevant. Today, people still enjoy laughing while watching Monty Python’s masterpiece. Thank goodness, it has not been banned, since we still live in a democracy.

**Figure 1:**
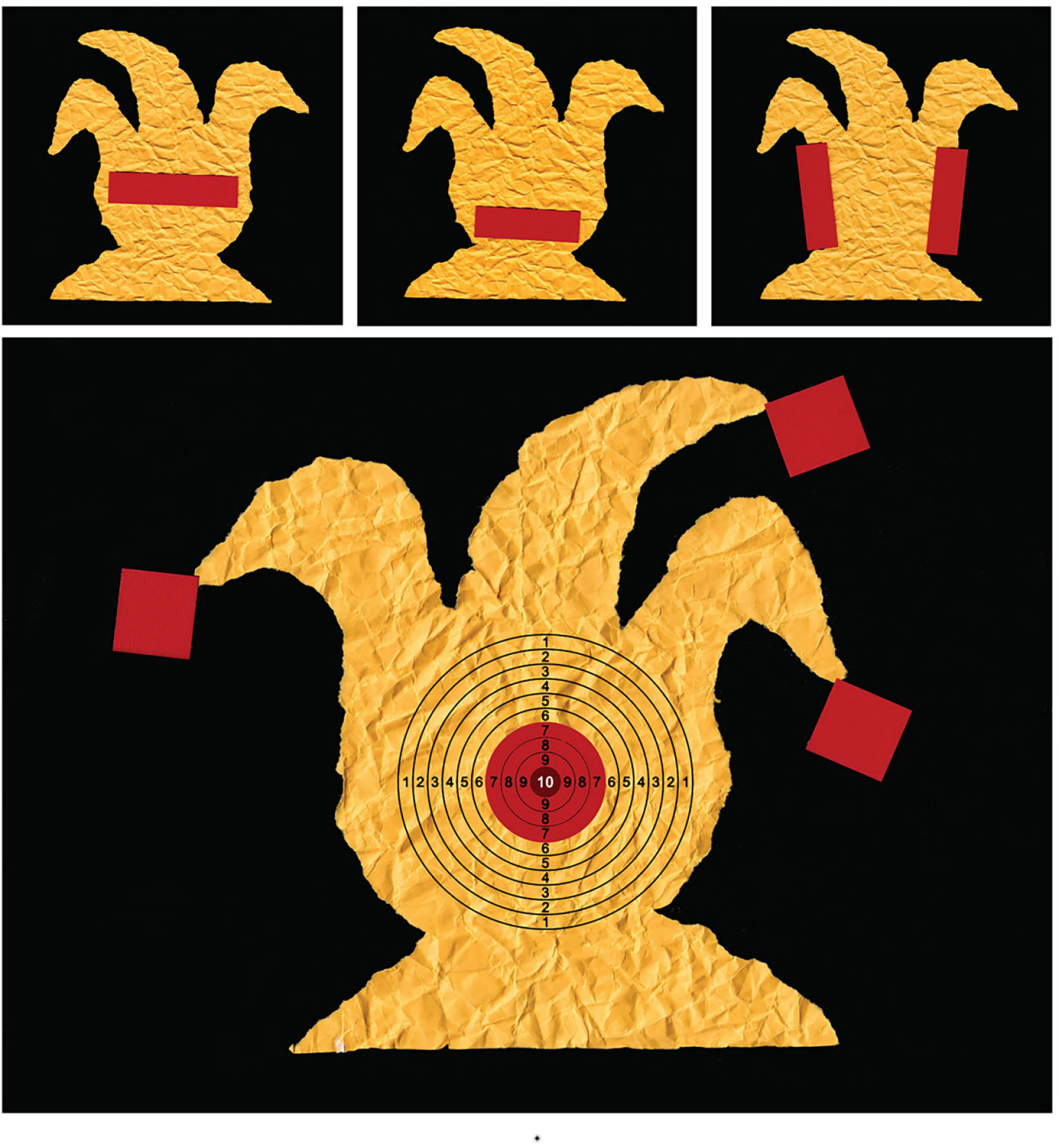
Fero Jablonovský, Clown-Monkeys. With permission from the artist.

But even today, in democracy, humourists do not have it easy (Fig. 1); the bishop’s role has now been taken over by the new agelasts. In the interview with Greg Lukianoff for FIRE (The Foundation for Individual Rights and Expression’s mission), John Cleese, one of the Monty Python’s founders, recognized the reactions to *The Life of Brian* back then in today’s Cancel Culture movement. He considers these people literal-minded who cannot cope with irony and sarcasm, who don’t get a metaphor, who like to have everything simple with the unbreakable rules. But they are also attempting to stop others from breaking the rules and this jeopardises not only humour but free speech generally. Other comedians such as George Carlin and Jerry Seinfeld have also been warning of the threat to the freedom of humour from political correctness and the perpetually offended. It’s not just totalitarian regimes where humour is suppressed; even in a democracy, we need to keep a close eye on the suppression of humour as a first sign of trouble.

## A sense of humour as a condition for living in truth

Comedian Július Satinský perceived humour as a worldview and writer Milan Kundera added that “the discreet light of humour uncovers the wide landscape of life.” Living with humour therefore means living in truth, a vision expressed by Václav Havel. In 1978, Havel wrote the famous essay *The Power of the Powerless*, which he dedicated to the memory of philosopher Jan Patočka, one of the main spokespersons of Charter 77 (Charta 77), the human rights movement in communist Czechoslovakia. Patočka died after brutal interrogations by the state police in 1977. Havel, the future president but at that time still a dissident against the totalitarian communist regime, predicted in that essay that totalitarianism would fall as soon as people have fulfilled their desire for dignity—for a life in truth. And that is what happened in 1989, when the people tore down the Iron Curtain and holloed: “Hello, Europe!”

A sense of humour was one of the fundamental pillars of Václav Havel’s life mission. He did not lose it even in communistic prison. His captors must have been furious when they read in his letters to his wife Olga, which were later published as a book, that he was definitely going to buy the new Bee Gees album. They failed to humiliate him.

But there is a fine line between comical and tragical, as Eugène Ionesco noted. In *The Power of the Powerless*, Havel warned that the violation of a humans’ moral integrity—the willingness to live in a lie—necessarily will lead to loss of freedom. And this is happening today: rejection of science, plundering of nature, collaboration with an aggressor. The Iron Curtain has been replaced by the Iron Net. But if life in truth was the power that broke down the Iron Curtain, then only life in truth can lead us out of the Iron Net. A necessary, but not sufficient, condition for this is not to lose our sense of humour.

Václav Havel did not lose his sense of humour, that rare gift of making fun of himself, even as president. In 1999, on the occasion of receiving the Open Society Prize awarded by Central European University’s in Budapest, he said: “Now, what should guide us? There are no exact guidelines. There are probably no guidelines at all. The only thing I can recommend at this stage is a sense of humour, an ability to see things in their ridiculous and absurd dimensions, to laugh at others and at ourselves. A sense of irony and everything that calls out for parody in this world. In other words, perspective or detachment. A sense for the hidden presence of all the most dangerous types of foolishness in others and in ourselves. A good mind. Unpretentious certainty about the meaning of things. Gratitude for the gift of life and the courage to take responsibility for it. Vigilance of the spirit. Who has not lost the ability to be aware of one’s own ridiculousness or insignificance is not arrogant and is not an enemy of an open society. Its enemy is a person with a grimly serious face and a burning gaze. I wish for all of us, and especially for the young students of Central European University, that we may preserve a cheerful mind, one of the basic tools of defence against madness and evil.”

## Supplementary information

Peer Review File
