# Supplementary material for: Humour uncovers the wide landscape of life: From laughter to freedom
Source: EMBO Rep. 2025 Jun 3;26(14):3474–7. doi: 10.1038/s44319-025-00494-6 (PMC12287520; doi:10.1038/s44319-025-00494-6)
Supplement: Supplementary file 1 — Peer Review File [file 44319_2025_494_MOESM1_ESM.pdf]

EMBO reports

Humour uncovers the wide landscape of life

Vladimir Leksa

Corresponding author(s): Vladimir Leksa (Vladimir.Leksa@savba.sk)

Review Timeline:

Submission Date:	29th Apr 25
Accepted:	27th May 25

Editor: Holger Breithaupt

Transaction Report: This article was editorially reviewed.
